# Predicting Radiotherapy Impact on Late Bladder Toxicity in Prostate Cancer Patients: An Observational Study

**DOI:** 10.3390/cancers13020175

**Published:** 2021-01-06

**Authors:** Francesco Catucci, Anna Rita Alitto, Carlotta Masciocchi, Nicola Dinapoli, Roberto Gatta, Antonella Martino, Ciro Mazzarella, Bruno Fionda, Vincenzo Frascino, Antonio Piras, Andrea D’Aviero, Francesco Preziosi, Giovanni Palazzoni, Vincenzo Valentini, Giovanna Mantini

**Affiliations:** 1 UOC Radioterapia Oncologica, Dipartimento di Diagnostica per Immagini, Radioterapia Oncologica ed Ematologia, Fondazione Policlinico Universitario “A. Gemelli” IRCCS, Largo Agostino Gemelli, 8, 00168 Roma, Italy; francesco.catucci@guest.policlinicogemelli.it (F.C.); annarita.alitto@policlinicogemelli.it (A.R.A.); carlotta.masciocchi@guest.policlinicogemelli.it (C.M.); nicola.dinapoli@policlinicogemelli.it (N.D.); antonella.martino@guest.policlinicogemelli.it (A.M.); ciro.mazzarella1@policlinicogemelli.it (C.M.); bruno.fionda@guest.policlinicogemelli.it (B.F.); vincenzo.frascino@policlinicogemelli.it (V.F.); giovanni.palazzoni@policlinicogemelli.it (G.P.); vincenzo.valentini@policlinicogemelli.it (V.V.); giovanna.mantini@policlinicogemelli.it (G.M.); 2 Dipartimento di Scienze Cliniche e Sperimentali dell’Università Degli Studi di Brescia, v.le Europa, 11, 25121 Brescia, Italy; roberto.gatta.bs@gmail.com; 3 Dipartimento Universitario di Scienze Radiologiche ed Ematologiche, Università Cattolica del Sacro Cuore, Largo Francesco Vito, 1, 00168 Roma, Italy; antoniopiras88@gmail.com (A.P.); francesco.preziosi@guest.policlinicogemelli.it (F.P.)

**Keywords:** decision supporting systems, predictive models, toxicity prediction, prostate cancer

## Abstract

**Simple Summary:**

Prostate cancer (PC) is the most common cancer in men over 70 years old, with heterogeneous characteristics and, nowadays, multiple treatment strategies obtaining optimal outcomes and improving survival. Personalized medicine and even more morbidity treatment reduction are increasing needs. The development of decision support systems to personalize treatments is a key challenge in oncology. The study PRODIGE 1.1 aimed to elaborate on a model analyzing dosimetric parameters in Prostate Cancer patients treating by radiation therapy, predicting late bladder toxicity in order to customize treatments and reduce late morbidity.

**Abstract:**

Background and purpose: The aim of our study was to elaborate a suitable model on bladder late toxicity in prostate cancer (PC) patients treated by radiotherapy with volumetric technique. Materials and methods: PC patients treated between September 2010 and April 2017 were included in the analysis. An observational study was performed collecting late toxicity data of any grade, according to RTOG and CTCAE 4.03 scales, cumulative dose volumes histograms were exported for each patient. Vdose, the value of dose to a specific volume of organ at risk (OAR), impact was analyzed through the Mann–Whitney rank-sum test. Logistic regression was used as the final model. The model performance was estimated by taking 1000 samples with replacement from the original dataset and calculating the AUC average. In addition, the calibration plot (Hosmer–Lemeshow goodness-of-fit test) was used to evaluate the performance of internal validation. RStudio Software version 3.3.1 and an in house developed software package “Moddicom” were used. Results: Data from 175 patients were collected. The median follow-up was 39 months (min–max 3.00–113.00). We performed Mann–Whitney rank-sum test with continuity correction in the subset of patients with late bladder toxicity grade ≥ 2: a statistically significant *p*-value with a Vdose of 51.43 Gy by applying a logistic regression model (coefficient 4.3, *p* value 0.025) for the prediction of the development of late G ≥ 2 GU toxicity was observed. The performance for the model’s internal validation was evaluated, with an AUC equal to 0.626. Accuracy was estimated through the elaboration of a calibration plot. Conclusions: Our preliminary results could help to optimize treatment planning procedures and customize treatments.

## 1. Introduction

Prostate cancer (PC) in Europe is the most common cancer in men over 70 years old [1], with approximately 449.761 new cases in Europe and 43.837 new cases in Italy in 2018 [2]. Despite its high prevalence, combining all stages, survival is high for PC (98%) [3].

Furthermore, prostate carcinoma is extremely heterogeneous, ranging from an indolent chronic illness to an aggressive, rapidly fatal systemic malignancy. The classic prognostic factors, tumor stage, prostate-specific antigen (PSA) level, and Gleason score (GS) have been combined to classify patients into distinct risk groups to determine the most appropriate treatment [4].

A multitude of treatments for PC patients, particularly in low-risk disease, is available [5,6,7]. Surgery, radiotherapy (RT), androgen deprivation therapy (ADT), active surveillance, or other therapeutic strategies, such as cryotherapy, high-intensity focused ultrasound, mini-invasive surgery, have offered advantages to patient survival even if sometimes resulting in worsening quality of life (QoL) and side effects [5,6,7].

For these reasons, new advances have been made in treatment modalities to reduce side effects while maintaining or improving outcomes.

This challenge has been met with new radiation technologies developed and adopted for clinical use in prostate cancer, responding to the need to deliver dose-escalated RT while minimizing treatment-related morbidity and toxicities.

In particular, RT techniques as intensity-modulated radiation therapy (IMRT) and technologies as image-guided radiation therapy (IGRT) and proton therapy contributed to improving outcomes and reducing toxicities. IMRT is able to deliver dose-escalated RT without increasing morbidity compared with 3-dimensional conformal radiotherapy (3D-CRT) at lower doses, so it is the recommended technique in PC by the European Association of Urology (EAU) [8] and the National Comprehensive Cancer Network (NCCN) [4].

Although many authors have demonstrated the ability of newer technologies than older ones to reduce organs at risk dose (IMRT vs. 3DCRT, proton vs. IMRT), there is a relative lack of studies directly comparing patient outcomes, including toxicities, QoL, and cancer control. Dose escalation (DE) improves disease control but could lead to increasing rectal and bladder side effects [9].

The best future standard of care will be the identification of “tailored” treatments based on specific cancer patients’ characteristics, both race differences and clinical aspects or side effects. The development of decision-making tools that allow delivering tailored treatment is crucial, in particular in cancer disciplines moving towards the era of individualized medicine [10,11].

The advances in diagnostic and treatment technology conducted a remarkable transformation in the internal medicine concept with the establishment of a new idea of personalized medicine. Inter- and intra-patient tumor heterogeneity, clinical outcomes’ and treatment toxicity’s complexity justifies the effort to develop predictive models (PMs) from decision support systems (DSSs) [12].

Identifying new indicators and developing models that could predict toxicity is important to choose the best treatment for patients [10,11,12,13].

The aim of this work was to evaluate the grade of genitourinary (GU) late toxicity by using IGRT and consequently to develop a preliminary model to predict toxicity. This purpose was realized by analyzing dosimetric parameters (the dose-volume histogram analysis of treatment plans) able to predict late bladder toxicity.

## 2. Materials and Methods

An observational study was performed through a retrospective analysis of patients treated between September 2010 and April 2017 at the Radiation Oncology Department of our Hospital.

Inclusion criteria were age ≥18 years, ECOG performance status ≤2, histologically proven PC), and no metastatic disease (cM0).

The majority of patients were treated with volumetric modulated arc therapy (vmat) through linear accelerator LINAC-6 MV photons. Clinical target volume (CTV) 1 (prostate) received a total dose of 80 Gy fractionated into 2 Gy/die. CTV2 (seminal vesicles—SV) received a total dose of 72 Gy, 1.8 Gy/die. In high-risk patients, CTV1 (prostate and SV) received a total dose of 67.5 Gy, 2.7 Gy/die, and pelvic lymph nodes (CTV2) received a total dose of 45 Gy, 1.8 Gy/die. Simultaneous integrated boost (SIB) technique was performed according to an internal schedule of treatment. A small number of patients were treated by sequential volume, still by volumetric arc therapy (RA), and the total dose varied from 73.8 Gy to 79.2 Gy to CTV1.

According to the risk class, the association with ADT was indicated [14].

Patients underwent MRI for correct staging [15] while CT images for planning were acquired with a conventional helical CT scanner (2.5 mm slices). Prostate, SV, bladder, rectum, femoral heads, penile bulb and small intestine, were manually contoured on the axial images in all CT scans, and the contours were verified by one physician. To ensure a homogeneous bladder filling and rectum emptying, patients were thought to drink 500 mL of water in 30 min and to perform a fleet enema before the simulation CT and every fraction. A vacuum bag immobilization system assured a supine position.

Cone beam CT scans imaging was performed during treatment for position monitoring.

Late bladder and rectal toxicity data were collected according to RTOG and CTCAE score [16,17]. The referring physician clinically examined patients a median of 5 times during RT treatment, unless otherwise requested by the patient himself. Dose volume histograms (DVH) pre and post-re-planning were analyzed for each organ at risk (OAR).

Dose from treatment planning and simulated delivery was evaluated using the equivalent uniform dose (*EUD*), as in the following equation, where *a* is a unitless model parameter that is specific to the normal structure, and *v_i_* is unitless and represents the *i*th partial volume receiving dose *D_i_* in Gy [18,19].

$$EUD=(\underset{i=1}{\sum }\left(v_{i}D_{i}^{a}\right)){}^{1/\alpha }$$

For normal tissues, the *EUD* represents the uniform dose, which leads to the same probability of injury as the examined inhomogeneous dose distribution. The *EUD* was calculated from the corresponding dose-volume distributions (histograms). The volume parameter found for bladder was *a* = 1.4 [20]. To calculate the *EUD*-based normal tissue complication probability (*NTCP*), we used parameterization of the dose-response based on logistic regression formalism [21,22]:

$$NTCP=\frac{1}{1+{\left[\frac{TD50}{EUD}\right]}^{4\gamma 50}}$$

where γ50 is the slope of sigmoidal dose-response curve of normal tissue at 50% complication probability.

$$=TD_{50}\cdot \;{\left|\frac{dNTCP}{dD}\right|}_{D=TD_{50}}$$

TCD_50_ is the tolerance dose for a 50% complication rate at a specific time interval when the wall organ of interest is homogenously irradiated.

A summary of numerical variables was reported as median, minimum and maximum values, while the categorical variables as percentages. We calculated the time of follow-up (FUP) from the start of RT to the last observation if the patient is still alive, otherwise until the time of analysis.

### Statistical Analysis

Data were analyzed by using R statistical software version 3.3.1 and by the in-house developed software package “Moddicom” [13,23]. A *p*-value cutoff of 0.05 was considered for significance. The value of dose to a specific volume of OAR (Vdose) impact on late GU toxicity was analyzed using a Mann–Whitney rank-sum test. Univariate logistic regression analysis was employed to identify independent predictors and the most significant Vdose value for late GU toxicity. Bladder DVHs have been analyzed using a univariate logistic model-based objective function, where the objective was to minimize the value of AIC according to Vdose variations. The Vdose value selection was performed by iterative search calculating the Akaike information criteria (AIC) for each logistic model. The dose value of the model with the lowest measure of AIC and a *p*-value less than 0.05 has been selected as the final Vdose parameter. The performance of this logistic model was evaluated using the area under the ROC curve (AUC). A calibration plot was elaborated in order to estimate the accuracy of the model using the Hosmer–Lemeshow goodness-of-fit (GOF) test: *p*-values < 0.05 indicate a lack of fit of the model. The entire dataset has been used for internal validation, employing a bootstrap resampling technique (TRIPOD type 1b internal validation [24]) to evaluate the predictive power of the model (PM).

In particular, 1000 datasets with the same size as the original one have been generated from primary patients by random sampling with replacement. The final value was the median measure of the AUC values, which were calculated for each dataset. A bootstrap method for internal validation was applied because this method is recommended in a case in which the sample size is limited, and the theoretical distribution of a statistic of interest is unknown, as in our case of study.

## 3. Results

### 3.1. Patients Characteristics

A total of 175 patients (mean age 72 years, min–max 53–86) were included in the study: 56.57% had T3a stage disease, 33.71% T3b, 8.57% had T2 disease (a and c), and 1.14% had stage T1 disease (a and b). All patients were clinically N0 and M0 (Table 1).

In 139 patients, a total dose of 80 Gy was prescribed on CTV1 (prostate) (mean dose 79.5; min–max 56–80; only three patients did not receive the prescribed dose for multiple comorbidities) delivered in 40 fractions within a median of 58 days (min–max 32–127); CTV2 (SV base or SV at all, respectively in cT3a or low stages and in cT3b) received a total dose of 72 Gy fractionated into 1.8 Gy/die [4].

In 21 high risks patients, CTV1 (prostate and base or whole SV, depending on the stage) received a total dose of 67.5 Gy fractionated into 2.7 Gy/die, and CTV2 (Pelvic nodes) received a total dose of 45 Gy fractionated into 1.8 Gy/die. SIB technique was used, according to internal schedules of treatment. Twelve patients were treated by sequential volume, still by VMAT, with a median total dose to CTV1 of 73.8Gy (min–max 73.8–79.2 Gy) and to CTV2 of 64.8 Gy (min–max 55.8–70.2). Only 3 patients were treated by IMRT, two with SIB technique, with a total median dose of 79.2 Gy (min–max 75.8–80), and to CTV2 of 70.2 Gy (min–max 70.2–72). All patients received ADT in neoadjuvant, concomitant and adjuvant (post-RT) settings in accord with their initial risk class [14], except seven for the initial favorable stage or for denial.The median FUP time was 39 months (min–max 3.00–113.00). All patients were still alive following the observations, except for one dead for causes not related to PC.

### 3.2. Analysis of Dosimetric Parameters Late Toxicity

Late toxicity data were collected in all patients. Late GU toxicity was G0 in 117/175 patients (66.85%), G1 in 48/175 (27.42%), G2 (in 10/175 (5.71%), G3 in 5/175 (2.8%), G4 (Severe hemorrhagic cystitis) in 1/175 (0.57%) (Table 2).

**Figure 1:**
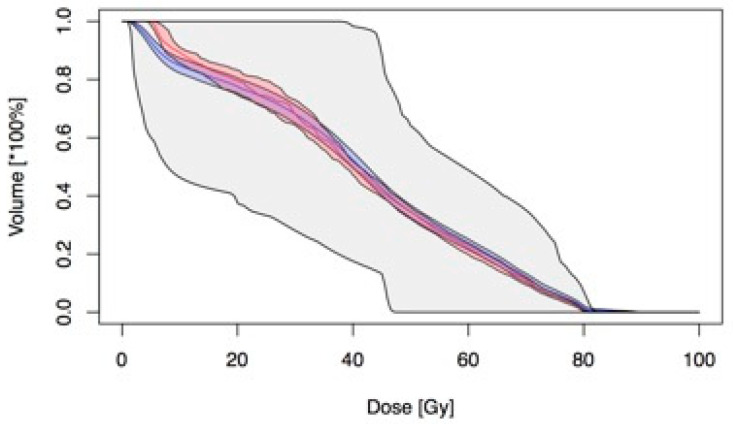
Summary of dose volume histograms (DVH) for nitourinary (GU) late toxicity with mean in blue and median in red and their relative inner confidence interval (IC).

Considering that the bladder could be assimilated as an OAR with a mixed “serial-parallel” radiobiological reaction [25,26], we first analyzed DVH (Figure 1).

Subsequently, we divided patients into two groups: a first group included patients without toxicity (G0); a second group included patients with any grade toxicity (G1, G2, G3, G4). Mean and median delivered doses of each group were compared. We performed the Mann–Whitney rank-sum test with continuity correction, and no statistically significant difference was found regarding GU toxicity.

**Figure 2:**
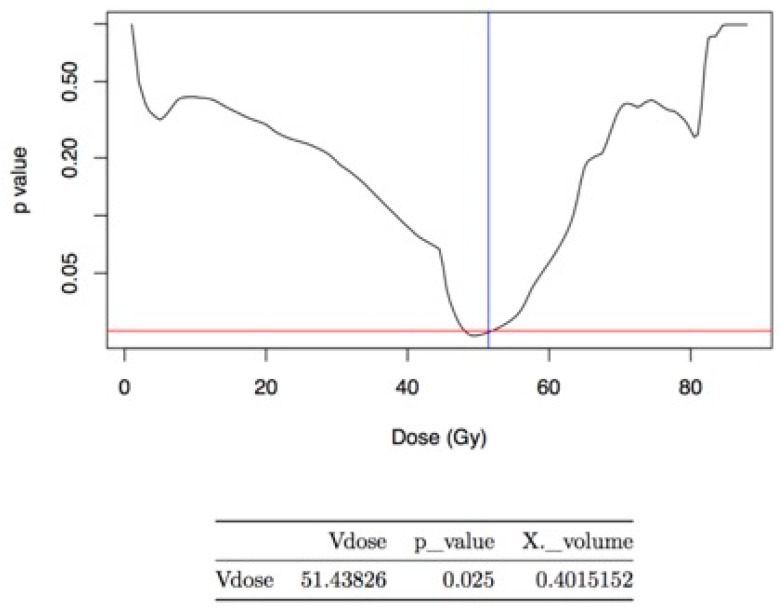
The figure shows the value of Vdose (equal to 51.43 Gy) as a predictor of the development of late G ≥ 2 GU toxicity.

We created a subset of patients (*n* = 58) from the second group, and we divided it into two other groups: the first included patients with GU toxicity of grade1 (G1); a second group included patients with GU toxicity G ≥ 2. In this subset, we recorded the lowest AIC score in case of late G ≥ 2 GU toxicity and a Vdose equal to 51.43 Gy (coefficient 4.3, *p* value 0.025) (Figure 2).

We also evaluated that the mean dose was higher in the group with late GU toxicity G ≥ 2, but without any statistical significance. Furthermore, a univariate logistic regression analysis was performed to evaluate the impact of clinical covariates (age at diagnosis, prostate dose, PSA at diagnosis and GS at diagnosis) on late G ≥ 2 GU toxicity, and no statistically significant differences were recorded.

**Figure 3:**
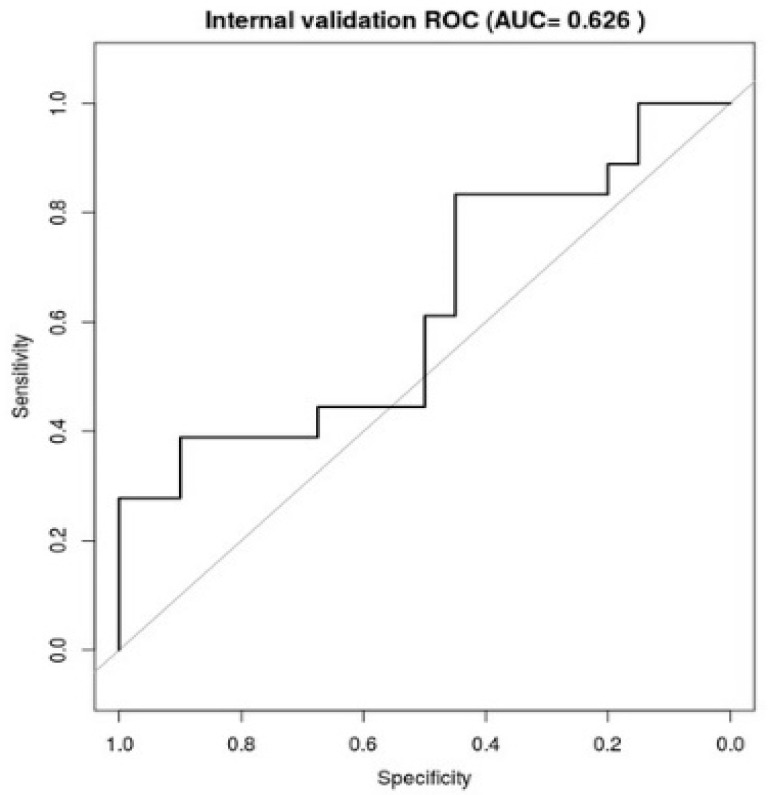
The figure shows a median of the Area under the ROC Curve (AUC) of the receiver operating characteristic curve (ROC) obtained for each dataset for internal validation.

The median of the AUC of the ROC curve obtained with an internal validation, using the bootstrapping technique (1000 random samples), was 0.626 (Figure 3).

**Figure 4:**
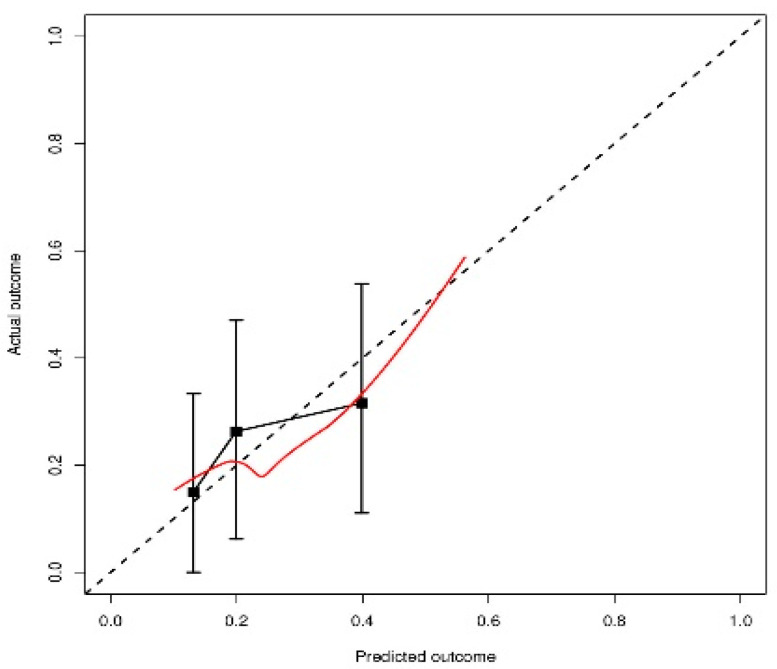
Calibration plot of Hosmer–Lemeshow goodness-of-fit (GOF) test (*p* > 0.05).

No statistically significant deviation was observed between the predicted and actual late G ≥ 2 GU toxicity (*p* > 0.05), as shown in Figure 4.

**Figure 5:**
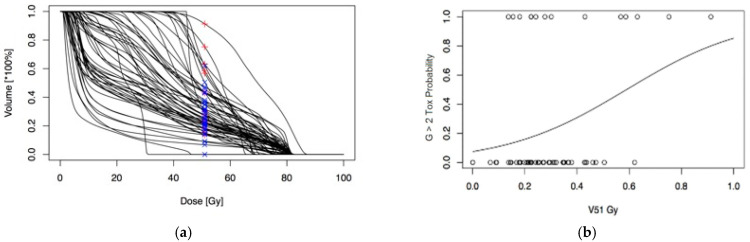
(**a**) Bladder volume (%) of each patient related to V51: in blue patients who have developed a GU toxicity = 1, in red patients who have developed a GU toxicity ≥ 2. (**b**) Plot with S slope that trims patients according to the risk of developing a GU toxicity > 2 in relation to V51.

Subsequently, by analyzing the data for patients who have manifested GU toxicity ≥ 2, we calculated the risk of developing such toxicity in relation to the percentage of bladder volume included in the isodose of 51.43 Gy (Figure 5a,b).

The following logistic regression formula was reported. In accordance with what was found in the analysis carried out, the *a* and coefficient *b* parameters of the model were −2.5 and 4.3, respectively.

$$P=\frac{e^{a+bX}}{1+e^{a+bX}}$$

From the analysis of this subset of patient data, we found that with a V51 < 8%, the risk of developing a GU toxicity ≥ 2 is <10%; with a V51 < 26.5%, the risk of developing a GU toxicity ≥ 2 is <20%; with a V51 < 59% the risk of developing a GU toxicity ≥ 2 is <50% (Table 3).

**Figure 6:**
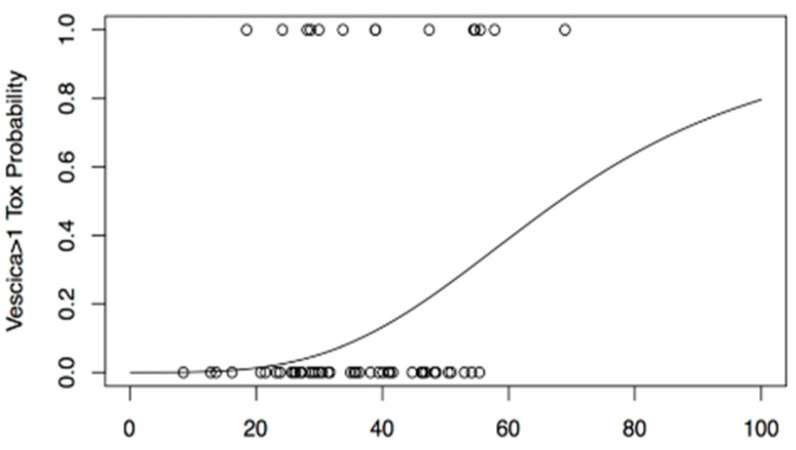
DVH-reduction model based on estimated complication probability (NTCP) under uniform irradiation (EUD) of the bladder.

Finally, we decided to use another dose-reduction method in order to simplify the evaluation of the toxicity that is the equivalent uniform dose (EUD), to elaborate a PM for GU toxicity with a grade G ≥ 2. We elaborated a DVH-reduction model by Niemierko, based on estimated complication probability (NTCP) under uniform irradiation (EUD) of the bladder. We found that our patients were distributed on or close to an S slope, showing that a 50% probability of late GU toxicity ≥ 2 is present with a mean delivered uniform dose of 68.04 Gy (TD50) (Figure 6).

## 4. Discussion

In recent years, the development of new treatments allowed to prolong survival in PC patients, sometimes with a worsening of QoL and side effects [5,6,7]. RT techniques were improved, allowing the safe administration of a higher dose of radiation [4]. IMRT is the preferred technique over 3D-CRT; although IMRT increases treatment costs [27], it appears to decrease rates of salvage therapy without a severe increase of side effects, especially when applied to patients with high-risk diseases [25]. Nowadays, the use of DE has become widespread, and many randomized trials have shown improved tumor control with the use of DE [28,29,30,31,32].

The difference in toxicity could be attributed to the combination of IMRT with dose reduction to OAR, daily image guidance and margin reduction [33]; these findings were also confirmed with patient reports through modified questionnaires [34]. IMRT reduced the delivery of significant radiation doses to bladder and rectum using a similar target volume, and it resulted in a lower risk of acute and late grade ≥ 2 GI and GU toxicity for IMRT compared with 3DCRT [35].

Our study, although with a small sample of 175 PC patients, analyzed the risk of late GU toxicity based on a cohort of patients undergoing RT. In our report, the grade of late toxicity was lower compared to GU late toxicity reported in the main studies of DE, both compared with the high dose group or conventional group [36,37]. Several DSSs have been developed in the last years related to different oncological diseases [12], even in predicting toxicity in PC [10]. Most available PMs focus on survival rates [38], which is only relevant to a limited patient population, but few models predicted adverse events.

Prediction of late bladder toxicity related to RT in PC or other pelvic tumors has yet been valued by other authors [39,40]. PMs can support the radiation oncologist to choose the most appropriate treatment also in relation to patient comorbidities.

In our study, we found a significant correlation between bladder G ≥ 2 late toxicity and a Vdose of 51 Gy, largely confirming the results shown in the QUANTEC report [33].QUANTEC report showed a consistent association between the volume of bladder receiving 51.4 Gy and the risk of grade ≥ 2 bladder; we calculated the risk of developing G ≥ 2 bladder toxicity in relation to the percentage of bladder volume included in the isodose of 51.43 Gy. An internal validation of the final model, using the bootstrapping technique, was performed to evaluate the performance, obtaining a median of the AUC of the ROC equal to 0.626.

We developed a mathematical model to study the bladder complication probability (NTCP), showing that a 50% probability of late G ≥ 2 late bladder toxicity is present with a mean delivered uniform dose of 68.08 Gy (Figure 5b).

A limit of our model is the flat distribution of every quartile response into the graph, confirming the heterogeneity of data sampling and evaluation and the large uncertainties raising from the small sample when dose-response relationship must be modeled. Another limitation of our study is the low outcome percentage and the inclusion of a single covariate (Vdose) in the final model since only one was statistically significant.

Furthermore, in order to confirm the strength of our model, it should be verified in a larger cohort of patients to increase the observation of patients with G ≥ 2 (especially for G = 3/4) and to effort the strength of the model.

Moreover, it would be preferred that the same physician evaluates the patient throughout the treatment period to have homogeneous data on toxicity grading and to standardize scores and pharmacological treatments for toxicity complications.

In the context of increasingly personalized oncology, it is necessary to build DSSs to obtain useful PMs to improve patients’ outcomes [10].

This road opens to new perspectives that provide the use of PMs combined with genomics and radiomics in order to implement tailor-made treatment. The development of tools that support the decision-making process to provide tailor-made treatment is crucial in the era of individualized medicine. A multidisciplinary and a multicentric collaboration are the key to develop accurate and scientifically based decision tools and obtain robust DSSs [10].

Clinical practice is usually based on evidence-based guidelines derived from randomized trials and meta-analyses with a homogenous enrolled population, but often, in daily routine, many patients may have different characteristics from those subgroups analyzed in trials, even because it is impossible to try every combination. Moreover, a long follow-up is needed for clinical trials, and their results may turn out to be outdated when finally published [10,38,41].

DSSs will be able to encompass the vision of classical clinical data and integrate a large number of heterogeneous features (i.e., information from diagnostic and treatment imaging, genomic and proteomic features, etc.), providing practical support to routinely clinical choices. By these interactions, guidelines and consensus could be enriched and completed by predictive models, avoiding patient over/under-treatment impacting on the costs. The cost-effectiveness analysis will be a crucial objective to better address resources [10,27,38,41].

DSSs need large heterogeneous databases to create a prediction model with a good statistical power underlying the importance of homogeneity of collected data and of a shared language [10]. The ontology-based methodology supports the creation of large databases [41]. Ontology “is a (formal) specification of concepts, relations and functions in a domain and hence focuses on concepts” [42].

A medical ontology is a model used to represent the concepts that compose the knowledge of a clinical domain; it contains all the relevant concepts related to a clinical field, organized in an explicit way that allows performing reasoning by automatic inference [10,43].

Following these principles, we are developing software able to predict the subjective toxicity developed by each patient in the context of increasingly participatory medicine. The final validation of the electronic PRO tool should allow helping to reduce and prevent complications of RT with a potential improvement in terms of PC patients’ QoL.

## 5. Conclusions

The development of decision support systems in order to reach “personalized treatments” is a challenging and even more actual aim. Our study seemed to encourage our hypothesis toward more personalized treatments in order to reduce complication rates and develop comparison multicentric studies with a large cohort of patients to customize treatments.

## Figures and Tables

**Table 1:** Patients and tumor characteristics.

Patients (*n*°)	175
Age (years; median (min–max))	72 (53–86)
T Stage (%)	
T1 (a and b)	1.14%
T2 (a and c)	8.57%
T3a	56.57%
T3b	33.71%
Gleason Score Grade Groups (*n*°)	
Grade 1	42.3%
Grade 2	29.7%
Grade 3	12.6%
Grade 4	11.4%
Grade 5	4%
PSA level (ng/mL (*n*°))	
<10	66.3%
10–20	22.3%
>20	11.4%
Androgen deprivation therapy (ADT (*n*°))	
Yes	96%
No	4%
Follow-up Median (months; median (min–max))	39.00 (3.00–113.00)

**Table 2:** Bladder late toxicity with score description.

Bladder Toxicity	RTOG Score	CTC Score	N. (%) TOT 175 Pts	Score Description
	G0		118 (67.43%)	Absent
	G1	1	43 (24.57%)	Mild frequency increasing microscopic hematuria
	G2	2	9 (5.14%)	Moderate frequency, intermittent hematuria
	G3	3	4 (2.29%)	Severe frequency and dysuria, frequent hematuria
	G4	4	1 (0.57%)	Severe hemorrhagic cystitis

**Table 3:** Risk of developing a GU toxicity ≥ 2 related to V51 (%).

V51 Volume (%)	Risk of GU Tox. ≥ 2
<8%	<10%
<26.5%	<20%
<59%	<50%

## Data Availability

The data presented in this study are available on request from the corresponding author.
